# Rapid and Sensitive LC-MS/MS Method for the Determination of Metoprolol in Beagle Dog Plasma with a Simple Protein Precipitation Treatment and Its Pharmacokinetic Applications

**DOI:** 10.3390/molecules17032663

**Published:** 2012-03-05

**Authors:** Sha Li, Xingli Wang, Kelong Peng, Zhiguo Ma, Xiaoqi Zhang, Shaolian Fu, Xiaofei Li, Linlin Li, Aihua Hong, Jie Jiang

**Affiliations:** 1 Department of Pharmaceutics, Jinan University College of Pharmacy, Guangzhou 510632, China; Email: tlisha@jnu.edu.cn (S.L.); roddick18@126.com (X.W.); mzg1979@126.com (Z.M.); liankefsl@163.com (S.F.); xiaofei.li2009@163.com (X.L.); daisy.lee920@163.com (L.L.); 2 College of Pharmacy, Tongji Medical School, Huazhong University of Science and Technology, Wuhan 430030, China; Email: sunpkl@163.com; 3 Guangdong Province Key Laboratory of Pharmacodynamic Constituents of TCM and New Drug Research, Jinan University College of Pharmacy, Guangzhou 510632, China; Email: xqzhang7401@yahoo.com.cn; 4 Jinan University Analytical and Testing Center, Guangzhou 510632, China; Email: hah_angel@126.com; 5 Institute of New Drug Research, Jinan University College of Pharmacy, Guangzhou 510632, China

**Keywords:** metoprolol tartrate, LC-MS/MS, pharmacokinetics, protein precipitation method

## Abstract

A rapid LC-MS/MS method with good accuracy and sensitivity was developed and validated for the pharmacokinetics study of metoprolol (MP) in beagle dogs. The plasma samples were simply precipitated by methanol and then analyzed by LC-MS/MS. An Ultimate XB-C_18_ column (150 × 2.1 mm *ID*, 5 μm) was used for separation, with methanol-water containing 0.2% formic acid (65:35, v/v) as the mobile phase at a flow rate of 0.2 mL/min. Monitoring ions of MP and internal standard (hydroxypioglitazone) were *m/z* 268.1/115.6 and *m/z* 373.1/150.2, respectively. The linear range was 3.03–416.35 ng**/**mL with an average correlation coefficient of 0.9996, and the limit of quantification was 3.03 ng**/**mL. The intra- and inter-day precision was less than 15%. At low, middle and high concentrations, the recovery, the matrix effect and the accuracy was in the range of 76.06%–95.25%, 93.67%–104.19% and 95.20%–99.96% respectively. The method was applied for the pharmacokinetics study of MP tartrate tablets (50 mg). The AUC_0-t_, T_max_ and C_max_ were respectively 919.88 ± 195.67 μg/L·h, 0.96 ± 0.33 h, 349.12 ± 78.04 ng**/**mL.

## 1. Introduction

Metoprolol (MP) is a kind of selective β_1_-adrenergic receptor blocker which has been widely used to treat hypertension, angina pectoris, arrhythmia, myocardial infarction and other cardiovascular diseases in the clinic. Nowadays, MP was used as different salt formats and as different dosage forms, such as conventional tablets, sustained release tablets, capsules and injections. The plasma concentration of MP and the β_1_ receptor antagonist activity showed a good correlation [1]. Moreover, research on novel formulations of MP have been increasing, for example, pulsatile-release tablets [2] and pellets [3]. The pharmacokinetics behaviour of MP in different dosage forms is important for providing reference to its clinical use and pharmaceutical formulation research.

The ultraviolet absorption of MP was relatively low, thus the HPLC-FL [4,5,6,7,8,9,10] method was often used for detecting MP in plasma. MP was also determined by GC-ECD [11,12]. Recently, the LC-MS/MS [13,14,15,16,17,18,19] method has been applied in some research to measure MP concentration in plasma due to the short analysis period and high selectivity. The research chiefly detected MP in human plasma, and no full validation of the methodology in dog plasma analysis was reported. In addition, the biological samples of MP were prepared mostly by the liquid-liquid extraction method [4,7,9,14,15,17], which is somewhat complicated. In this work, we treated the plasma samples by a simple protein precipitation method and developed a fast, sensitive and accurate LC-MS/MS method for quantitative analysis of MP in beagle dogs. The method was fully validated and subsequently applied to the pharmacokinetics study of MP tartrate tablets to verify its availability in the non-clinical pharmacokinetics study of MP formulations.

## 2. Results and Discussion

### 2.1. LC-MS/MS Analysis

**Figure 1:**
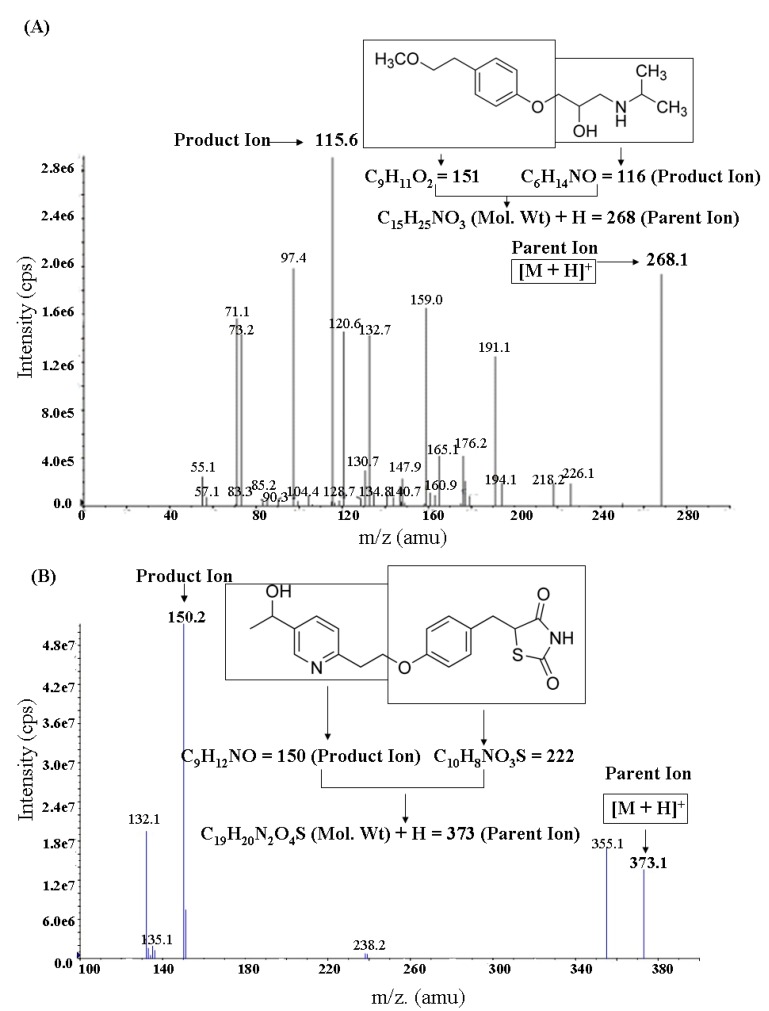
Profile mass spectral data of (**A**) metoprolol [M+H]^+^ and (**B**) hydroxypioglitazone [M+H]^+^.

Full-scan was conducted separately in both positive and negative ion mode. It was found that the response of the parent ion in positive ion mode was much higher than that in negative ion mode for both MP and IS. The ionization of the analytes was carried out using ESI in the positive mode. The protonated molecule ion peaks ([M+H]^+^) of MP and IS were observed at *m/z* 268.1 and *m/z* 373.1, respectively, in unit scan. After fragmentation in the collision cell, the fragment ion of highest response was selected as the product ion for monitoring MP (*m/z* 115.6) and IS (*m/z* 150.2) (Figure 1).

**Figure 2:**
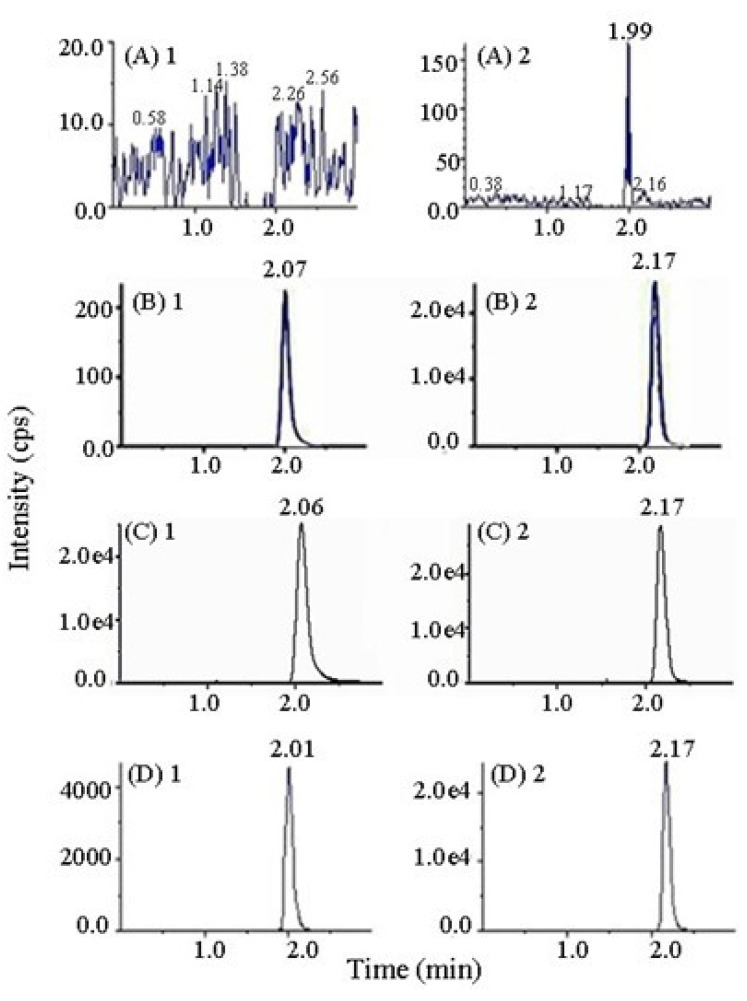
Typical chromatograms of (**A**) drug-free plasma; (**B**) drug-free plasma spiked MP (3.03 ng/mL) and IS (86.40 ng/mL); (**C**) drug-free plasma spiked MP (416.35 ng/mL) and IS (86.40 ng/mL); (**D**) plasma sample obtained at 4 h after a single oral administration of MP tartrate tablets (50 mg). 1: MP; 2: IS.

In this work, formic acid was added to the mobile phase to improve the peak tailing of MP. The retention times of MP and IS were about 2.06 min and 2.17 min separately (Figure 2). The LC-MS/MS method finished the analysis of each sample within 3.0 min. The method was thus suitable for analyzing a relatively large number of samples in short time.

### 2.2. Preparation of Plasma Samples

For measuring MP in plasma samples, the samples were prepared mostly by the liquid-liquid extraction method [4,7,9,14,15,17]. In this work, a simpler protein precipitation method with methanol was applied to prepare plasma samples, which simplified the operation and used less plasma volume as well. In addition, proteins were completely precipitated by using four-time volume of methanol which provided good protection of the column.

### 2.3. Method Validation

#### 2.3.1. Selectivity

The selectivity of LC-MS/MS method was investigated by comparing chromatograms of drug-free plasma, drug-free plasma spiked with MP and IS, and plasma samples obtained after drug dosing. Good separation and symmetric peak shape was showed in Figure 2. And no endogenous components in beagle dog plasma interfered with the determination.

#### 2.3.2. Linearity and Lower Limit of Quantification

The calibration curve was process by least squares linear regression (w = 1/x^2^). A good linear relationship was showed between the peak area ratio of MP/IS and MP concentration within the range of 3.03–416.35 ng/mL. The average correlation coefficient (r) was 0.9996 ± 0.0001 (n = 5). The average equation of calibration curves was 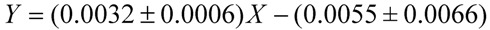 (n = 5). The lower limit of quantification (LLOQ) was 3.03 ng/mL. The precision and accuracy for the LLOQ was 8.72% and 99.96% ± 8.71% respectively (Table 1), which was acceptable for the analysis of biological samples.

#### 2.3.3. Precision and Accuracy

The results of precision and accuracy were listed in Table 1. The intra-day and inter-day precision, respectively, ranged from 2.54% to 10.65% and from 5.01% to 8.51%, and the accuracy ranged from 95.20% to 99.96%. Both were within the acceptable ranges for analysis of biological samples, ≤15% for precision and 85%–115% for accuracy.

**Table 1:** Precision and accuracy of MP in plasma (n = 5).

Concentration Added (ng/mL)	Concentration Observed (ng/mL)	Intra-day RSD (%)			Inter-Day RSD (%)	Accuracy (%)
		First Day	Second Day	Third Day		
3.03	3.03 ± 0.26	8.72	-	-	-	99.96 ± 8.71
6.06	5.84 ± 0.29	2.59	5.57	5.15	5.01	96.40 ± 4.82
181.68	172.96 ± 10.20	3.01	4.50	2.54	5.89	95.20 ± 5.61
363.36	347.91 ± 29.62	10.65	10.44	2.78	8.51	95.75 ± 8.15

#### 2.3.4. Recovery and Matrix Effect

The recovery and matrix effect results were summarized in Table 2. At low, middle and high concentration, the recovery of MP was 76.06%, 93.41% and 95.25% respectively, and that of IS was 93.71%. Although the recovery of MP at low concentration was not as high as that at higher concentration, the results were reproducible and consistent with a RSD of 4.45%. The recovery of MP at middle and high concentration and that of IS was also showed small variation with the RSD less than 3.07%. Therefore, the simple protein precipitation method was proved to be efficient enough to separate and extract MP and IS simultaneously from beagle dog plasma.

**Table 2:** The results of recovery and matrix effect (n = 5).

Drug	Concentration Added (ng/mL)	Recovery (R)		Matrix Effect (ME)	
		R (%)	RSD (%)	ME (%)	RSD (%)
MP	6.06	76.06 ± 3.38	4.45	93.67 ± 8.88	9.48
	181.68	93.41 ± 2.47	2.64	100.85 ± 6.27	6.22
	363.36	95.25 ± 2.93	3.07	104.19 ± 5.71	5.48
IS	86.40	93.71 ± 2.29	2.44	97.54 ± 2.15	2.20

The endogenous components are the main cause of ion suppression effects during electrospray ionization. The extent of this effect is chiefly affected by the sample extraction procedure and is compound dependent as well [15]. The results showed that the matrix effect of MP ranged from 93.67% to 104.19% and that of IS was 97.54%. It followed that no endogenous matrix components interfered with the ionization of the analytes.

#### 2.3.5. Stability

The results of stability of samples exposed to different experimental conditions are shown in Table 3. The deviation of the observed concentration of samples from the nominal concentration was less than ±6%, and the variation was less than 10% in RSD under all testing conditions. The results indicated that MP was stable in both untreated and post-preparative beagle dog plasma samples over all steps of the analysis.

**Table 3:** The results of stability test (n = 3).

Conditions	Concentration Added (ng/mL)	Concentration Observed (ng/mL)	Accuracy (%)	RSD (%)
Long-term ( two months)	6.06	5.97 ± 0.48	98.49 ± 7.91	8.03
	181.68	177.06 ± 4.73	97.46 ± 2.60	2.67
	363.36	354.33 ± 6.27	97.52 ± 1.72	1.77
Short-term (24 h)	6.06	6.18 ± 0.23	102.13 ± 3.80	3.69
	181.68	177.24 ± 1.60	97.56 ± 0.89	0.91
	363.36	360.98 ± 26.25	99.30 ± 7.25	7.27
Frozen-thaw	6.06	5.75 ± 0.36	95.01 ± 5.94	6.19
	181.68	171.11 ± 8.31	94.18 ± 4.57	4.86
	363.36	342.22 ± 9.13	94.18 ± 2.51	2.66
Post-preparative (24 h)	6.06	5.74 ± 0.35	94.77 ± 5.87	6.16
	181.68	176.92 ± 1.80	97.51 ± 1.22	1.02
	363.36	354.17 ± 7.67	97.47 ± 2.11	2.17

### 2.4. Pharmacokinetics Study of MP Tartrate Tablets in Beagle Dogs

**Figure 3:**
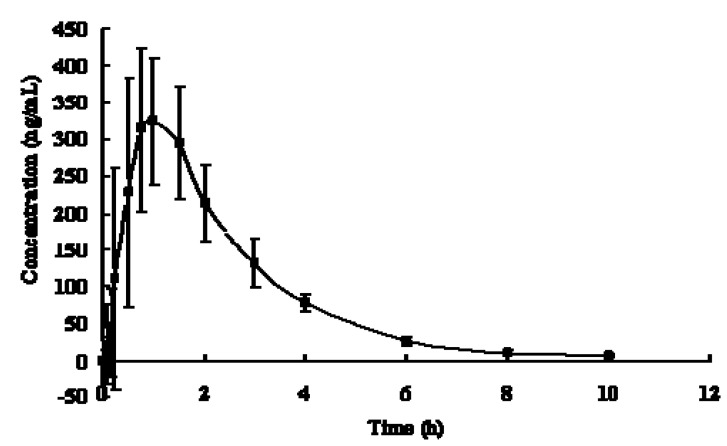
Mean plasma concentration-time profiles for MP in Beagle dogs after a single oral administration of MP tartrate tablets (2 pieces, 50 mg) (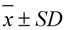, n = 6).

The LC-MS/MS method was successfully applied to the pharmacokinetics study of MP tartrate tablets in beagle dogs. The plasma concentration-time curve after a single oral dosing was illustrated in Figure 3. After oral administration, the concentration of MP fast reached the peak concentration (349.12 ± 78.04 ng/mL) within 1 h, followed by a slow drop thereafter. The main pharmacokinetics parameters were obtained by processing the data with DAS software and summarized in Table 4. In this work, T_max_ and t_1/2_ were calculated as 0.96 ± 0.33 h and 1.73 ± 0.49 h respectively, whichwere close to the previous reported values [5,13,14,20]. However, AUC and C_max_ were not matched so much with the reported values due to the different dosage form, administration dose, blood sampling time point and individual difference.

**Table 4:** The main pharmacokinetic parameters after a single oral administration of MP tartrate tablets (50 mg) in Beagle dogs (n = 6).

Pharmacokinetic Parameters	Mean ± SD
AUC_0-t_ (µg/L·h)	919.88 ± 195.67
AUC_0-∞_ (µg/L·h)	934.42 ± 200.92
MRT_0-t_ (h)	2.40 ± 0.19
MRT_0-∞_ (h)	2.56 ± 0.17
t_1/2_ (h)	1.73 ± 0.49
T_max_ (h)	0.96 ± 0.33
C_max_ (ng/mL)	349.12 ± 78.04

## 3. Experimental

### 3.1. Drugs and Reagents

Metoprolol tartrate tablets were obtained from AstraZeneca Company Ltd. (trade name Betaloc, Lot: 1003035, 25 mg/tablet, Sweden); Metoprolol (MP) tartrate reference standard was supplied by National Institutes for Food and Drug Control (Lot: 100084-200101, China); Hydroxypioglitazone (Internal standard, IS) was provided by Toronto Research Chemicals Inc. (Lot: 5-XAL-112-3, Canada); Methanol of chromatography grade was obtained from Fischer Company Ltd. (Lot: 101437, USA); All other chemicals were analytical grade.

### 3.2. Animals

Beagle dogs (male, 10 ± 2 kg) were supplied by Kangda Experimental Animal Technology Company Ltd., Guangdong, China. The animals were kept in an environmentally controlled breeding room and fed with standard laboratory food and water *ad libitum*.

### 3.3. LC-MS/MS Conditions and Instrumentation

An Agilent 1200 series HPLC system (Agilent Technologies Company, USA), consisting of G1311A quaternary pump, G1329A autosampler and G1316A column oven, was used for separation. The HPLC was coupled to a triple quadrupole tandem mass spectrometer (API 4000^TM^ LC-MS/MS system, Applied Biosystems Company, USA) with an electrospray ionization (ESI) interface. Chromatography workstation Analyst 1.5 (Applied Biosystems Company, USA) was used for data acquisition and processing.

Chromatographic conditions: Chromatographic separation was achieved on Ultimate XB-C_18_ column (150 × 2.1 mm *ID*, 5 μm, Welch Materials Inc., USA) with a SecurityGuard C_18_ guard column (4.0 × 3.0 mm *ID*, 5 μm, Phenomenex, USA). The mobile phase consisted of methanol and water containing 0.2% formic acid (65:35, v/v). The flow rate was 0.2 mL/min. The injection volume was 5 μL. The column temperature and the injector temperature were maintained at 35 °C and 15 °C respectively. The total analysis time was 3 min.

Mass spectrometry conditions: The tuning parameters of mass spectrometry were optimized by injecting 500 ng/mL of standard solution containing MP and IS at 10 μL/min by means of an external syringe pump directly connected to the mass spectrometer. Full-scan was conducted in both positive and negative ion mode, separately. The ionization of the analytes was carried out using ESI in the positive mode. MP and IS were respectively quantified using transitions of *m/z* 268.1/115.6 and *m/z* 373.1/150.2. The optimal working parameters were as follows. The ion spray source gas temperature (TEM) was set at 500 °C. The ion spray voltage (IS) was set at 5,500 V. The nebulizer gas (GS1), turbo ion spray gas (GS2) and the curtain gas (CUR) values were set at 40, 60 and 25 psi, respectively. The collision associated dissociation gas (CAD) value was fixed at 5 psi. Declustering potential (DP), entrance potential (EP), collision energy (CE), collision cell exit potential (CXP) were respectively 78 V, 10 V, 26.4 V, 13 V and 110 V, 10 V, 40 V, 13 V for MP and IS.

### 3.4. Preparation of Standard and Quality Control Working Solutions

MP tartrate was dissolved in methanol to prepare a standard stock solution of 75.7 μg/mL (calculated as MP). The stock solution was diluted further to obtain standard working solutions (4,163.5, 2,271.0, 1,135.5, 605.6, 227.1, 113.6, 30.3 ng/mL) and quality control (QC) working solutions (60.56, 1,816.8 and 3,633.6 ng/mL). Hydroxypioglitazone was dissolved in chloroform of an appropriate volume, and diluted further with methanol to yield a solution of 864 ng/mL. All solutions were stored at 4 °C.

### 3.5. Preparation of Plasma Samples

IS solution (20 µL) and methanol (20 µL) were added into each plasma sample (200 µL) and vortex for 10 s, then 760 µL of methanol was added again and vortex for 30 s. The mixture was centrifuged at 13,000 rpm for 10 min to remove precipitate. The supernatant (200 µL) was measured and added with 0.25% formic acid solution (50 µL), and then injected into LC-MS/MS system after mixing well.

### 3.6. Method Validation

#### 3.6.1. Selectivity

To confirm lack of interference and absence of variation, the selectivity of the method was evaluated by analyzing drug-free plasmas, drug-free plasmas spiked with MP (at 3.03 and 416.35 ng**/**mL) and IS, and plasma samples obtained after administration of MP tartrate tablets from six different beagle dogs.

#### 3.6.2. Linearity and Lower Limit of Quantification

A series of drug-free plasma (200 µL) was spiked with 20 μL of MP standard working solutions, to prepare plasma standard samples of different MP concentrations (416.35, 227.10, 113.55, 60.56, 22.71, 11.36, 3.03 ng/mL). The plasma standard samples were treated and analyzed as described previously. The peak area of MP and IS was recorded and the peak area ratio of MP/IS was calculated. The plasma calibration curve was plotted by the peak area ratios of MP/IS versus the corresponding concentrations of MP. The plasma calibration curve was prepared again daily.

The lower limit of quantification was determined as the lowest concentration on the calibration curve that could be quantified with a signal-to-noise ratio of at least 10 and acceptable precision (≤20%) and accuracy (80%–120%).

#### 3.6.3. Precision and Accuracy

The QC samples of MP were prepared by the same method as plasma standard samples with QC working solutions, and the low, middle and high concentrations were set as 6.06, 181.68 and 363.36 ng/mL respectively. The intra-day precision was determined in 5 replicates of QC samples, respectively at low, middle and high concentration, on the same day. The inter-day precision and accuracy was measured on 3 separate days, 5 replicates of QC samples respectively at low, middle and high concentration for each day.

The precision was expressed as percentage of relative standard deviation (RSD%). RSD% was calculated according to formula (1), where 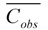 is the average observed concentration of QC samples. The accuracy was calculated by comparing the observed concentration (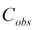) with the nominal concentrations (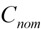) of analytes in QC samples according to formula (2):


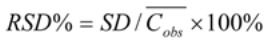
(1)


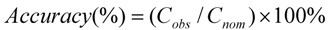
(2)

#### 3.6.4. Recovery and Matrix Effect

The recovery and matrix effect was determined using five replicates at three QC concentrations separately. The recovery was calculated by comparing the peak areas of MP and IS in QC samples with the average peak areas of analytes in samples of the same concentrations which were prepared by spiking the analytes into drug-free plasma after protein precipitation. The matrix effect was determined by comparing the peak areas of MP and IS spiked into deproteinized drug-free plasma with the average peak areas of analytes in neat solutions of the same concentrations.

#### 3.6.5. Stability

The stability of MP in plasma was assessed by analyzing three replicate QC samples, at low, middle and high concentration respectively, exposed to different time and temperature conditions, including long-term storage at −80 °C for two months, short-term storage at room temperature for 24 h, and three frozen-thaw cycles treatment. Also, the post-preparative stability of MP was evaluated with the processed QC samples stored at 15 °C in an autosampler for 24 h. The concentrations measured from the above samples were compared with the nominal values of QC samples. Samples were concluded stable and applicable for routine analysis if the deviation of observed sample concentration was less than ±15% from the nominal value.

### 3.7. Pharmacokinetics Study of MP Tartrate Tablets in Beagle Dogs

The animal study was approved and performed in accordance with the guidelines of Institutional Animal Ethics Committee. Six beagle dogs were fasted for 12 h before dosing. Each animal was administered two pieces of MP tartrate tablets (25 mg/tablet). Blood samples (2 mL) were collected from forelimb vein into heparinized tubes before administration (0 min), and at 5, 10, 15, 30, 45, 60, 90, 120, 180, 240, 360, 480 and 600 min after administration. The blood samples were centrifuged at 4,000 rpm for 10 min, and the plasma were transferred and stored at −80 °C until analysis. In analysis, the samples of concentration above the analytical range was diluted by blank plasma and then measured again, The samples of concentration below the analytical range was not included in the calculation.

The data were processed by DAS (Drug and Statistics) computer software package program (Version 3.0.1, Sun Ruiyuan, Chinese Pharmacology Society, China) and the pharmacokinetic parameters were calculated by moment analysis. The maximum plasma concentration (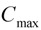) and the time to reach 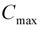 (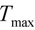) were estimated directly from the experimental observation values. The area under the plasma concentration-time curve from time zero to time of the last measurable concentration (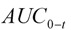) was calculated by the trapezoidal rule. The AUC extrapolated to infinity (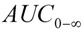) was calculated by formula (3), in which 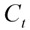 is the last measurable plasma concentration, 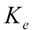 is the terminal elimination rate constant. The 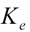
> was calculated from the slope of the terminal logarithm plot of plasma concentration versus time curve using linear least-squares regression method. The terminal elimination half-life (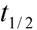) was calculated by formula (4):


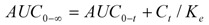
(3)


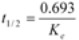
(4)

## 4. Conclusions

A fast, sensitive LC-MS/MS method of high selectivity, precision, recovery and accuracy was developed to measure MP concentration in beagle dog plasma and fully validated. The method has a high throughput capability because of the simple sample preparation by protein precipitation and short total analysis time within 3 min. The LC-MS/MS method was successfully applied to the pharmacokinetic study of MP tartrate tablets in beagle dogs. The method may provide valuable reference for the pre-clinical pharmacokinetic study of other formulations of MP in dogs or other animals, which are indispensable for the formulation research and development of MP. Moreover, with appropriate validation, the method may be extended to determine the concentration of MP in human plasma and thus applied to clinical pharmacokinetics studies as well.
